# Supplementary material for: Comparative Structure Analysis of the Multi-Domain, Cell Envelope Proteases of Lactic Acid Bacteria
Source: Microorganisms. 2023 Sep 8;11(9):2256. doi: 10.3390/microorganisms11092256 (PMC10535647; doi:10.3390/microorganisms11092256)
Supplement: Supplementary file 1 [file microorganisms-11-02256-s001.zip › microorganisms-2554342-supplementary.pdf]

Supplementary Materials

Comparative structure analysis of the multi-domain, cell envelope proteases of lactic acid bacteria

Lise Friis Christensen[1,*], Magnus Haraldson Høie[2], Claus Heiner Bang-Berthelsen[1], Paolo Marcatili[2], and Egon Bech Hansen[1,*]

[1] National Food Institute, Technical University of Denmark, Kemitorvet, DK-2800 Kgs. Lyngby, Denmark
[2] Department of Health Technology, Technical University of Denmark, Ørsteds Plads, DK-2800 Kgs. Lyngby, Denmark
* Corresponding authors

Content of supplementary material

Figure S1. PrtP protein homologs from plant derived lactic acid bacteria (LAB) strains.
Figure S2. Phylogenetic three of PrtP protein homologs based on their whole protein sequences.
Figure S3. Predicted aligned error (PAE) plots for AlphaFold 2 (AF) models.
Figure S4. Multiple sequence alignment (MSA) depth for AlphaFold 2 (AF) models.
Figure S5. The predicted local distance difference test (pLDDT) for AlphaFold 2 (AF) models.
Figure S6. Intramolecular comparison of fibronectin type-III-like (Fn) domains.
Figure S7. Long tandem repeats in the cell wall spacing (W) domain.

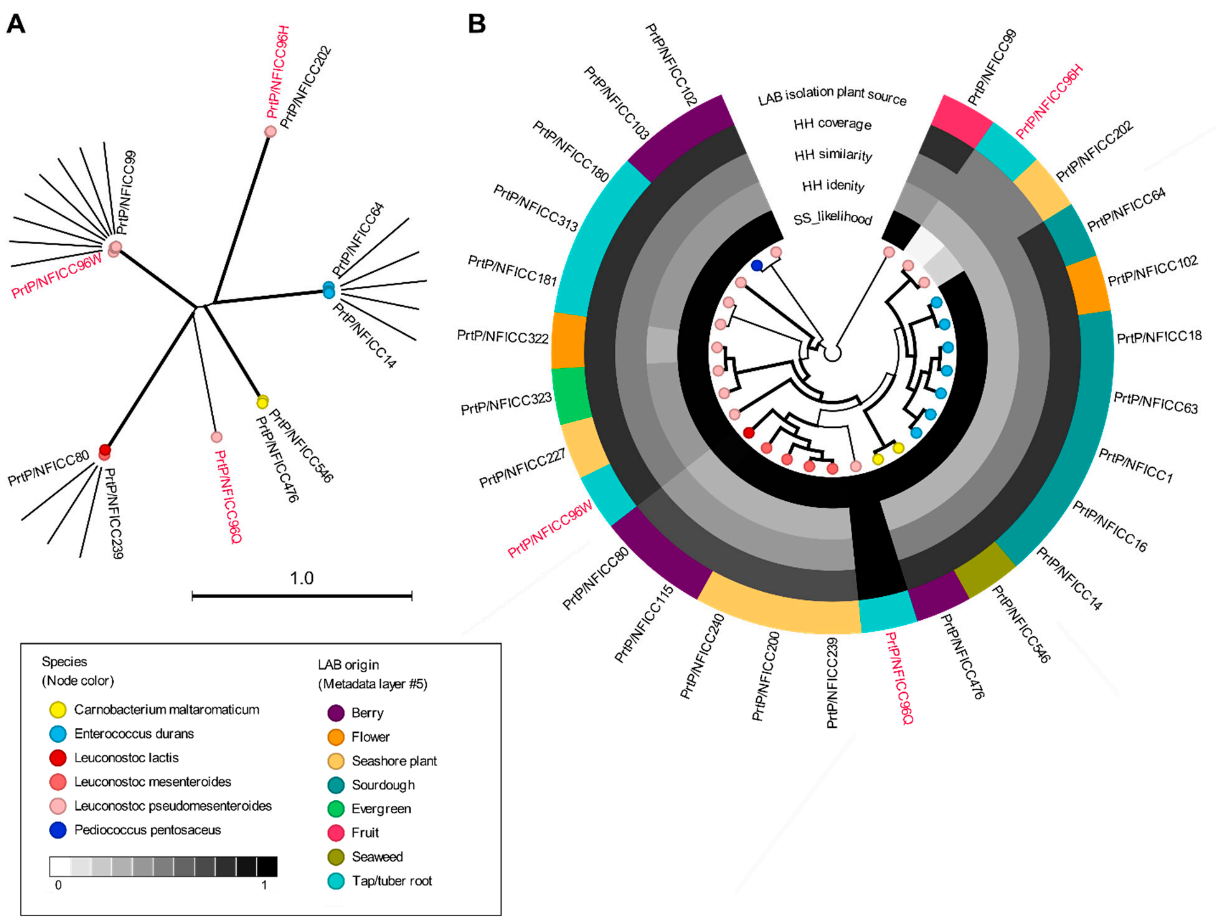

Figure S1. PrtP protein homologs from plant derived lactic acid bacteria (LAB) strains.
The entire PrtP homologous protein sequences were used to build a phylogenetic tree, with the tree topography determined by the neighbor-joining method. Bootstrapping with 100 replicates was used to assess tree reliability. Most branches were supported by bootstrapping above 70%, as shown by the thick lines. The phylogenetic tree is shown as a radial tree (A) and a circular cladogram with metadata layers (B). The PrtP homologs were discovered using genome sequences of LAB strains derived from various plant isolates. The LAB strains were from the National Food Institute Culture Collection (NFICC), and the proteases were named PrtP, followed by the name of the LAB strain. Three PrtP homologs (red) were found in a single LAB strain, with names including the last letter of the corresponding contig names. The LAB strains included different species (node color). The black branch lengths of the radial phylogenetic tree were proportional to the Jukes-Cantor distances, as indicated by the scale bar. The circular cladogram showed five metadata layers #1 – #5. The metadata layer #1 to #4 were colored according to the gray scale bar values. Metadata layer #1 displayed the predicted likelihood for a secretory signal (SS) peptide, whereas metadata layer #2, #3 and #4 displayed the protein sequence identity, similarity and coverage of pairwise HHpred comparisons between $PrtP_{Wg2}$ and each of listed PrtP homologs.

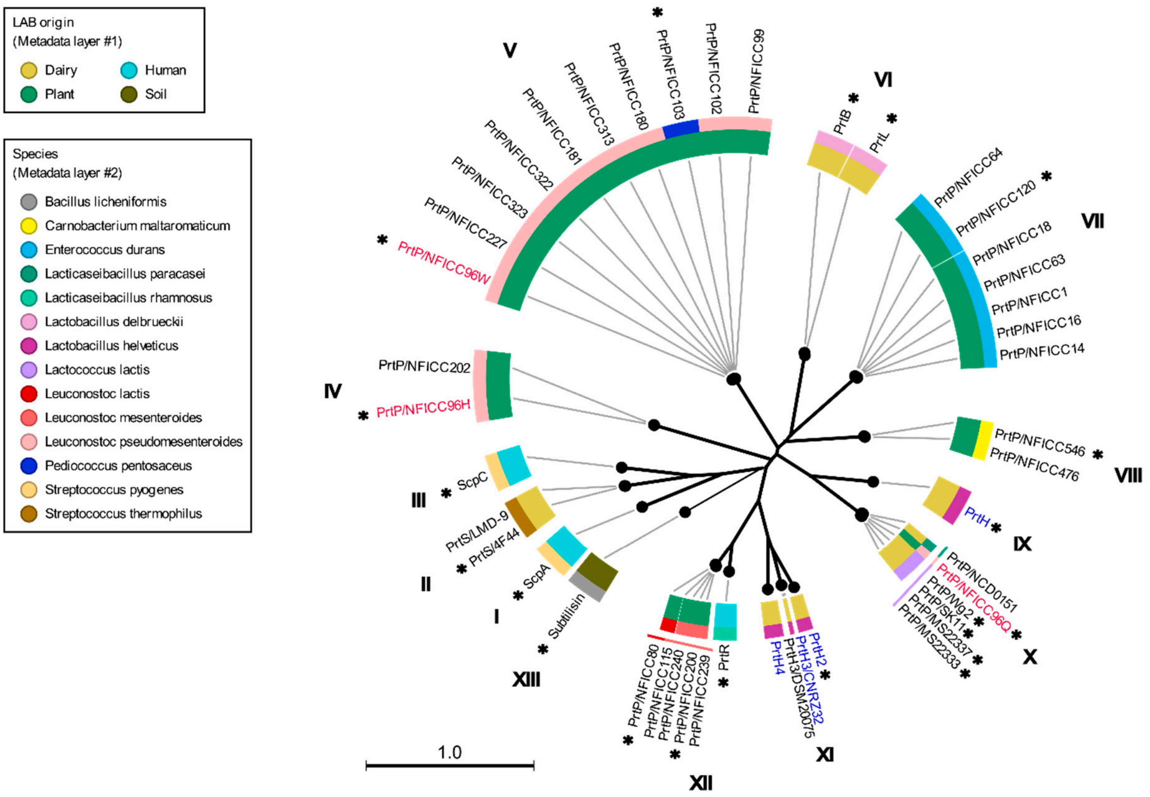

Figure S2. Phylogenetic three of PrtP protein homologs based on their whole protein sequences.
The entire protease sequences were used to construct the radial phylogenetic tree. The names of the LAB strains were provided as part of the protease name to distinguish between the proteases with the same name. The LAB strains derived from plant sources were from the National Food Institute Culture Collection (NFICC). The neighbor-joining method was used to generate the tree topography. Black branch lengths were proportional to the Jukes–Cantor distances, indicated by the scale bar. Bootstrapping with 100 replicates was used to assess the tree reliability. All branches were verified with bootstrapping above 70 %, as shown by the thick lines. PrtP homologs were found in different bacteria strains of different species (outer circle layer) that were derived from different origins (inner circle layer). Three PrtP homologs (red) were from *Leuconostoc pseudomesenteroides* NFICC96 like the four PrtP homologs (blue) were from *Lactobacillus helveticus* CNRZ32. The clusters of PrtP homologs were assigned I – XIII based on the phylogenetic tree in figure 1, but follow the same order in this tree, counting clockwise after subtilisin. PrtP homologs marked with asterisks were included for further structural analysis.

PrtS_{4F44}

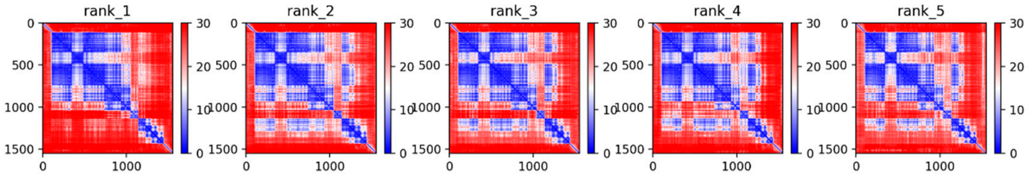

$\text{PrtP}_{NFICC96H}$

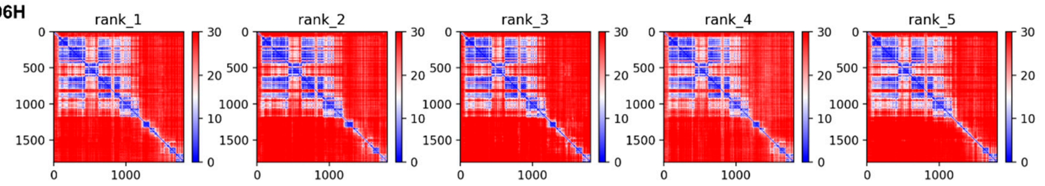

$\text{PrtP}_{NFICC96W}$

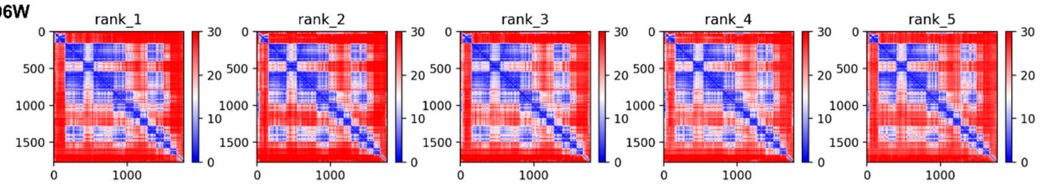

$\text{PrtP}_{NFICC103}$

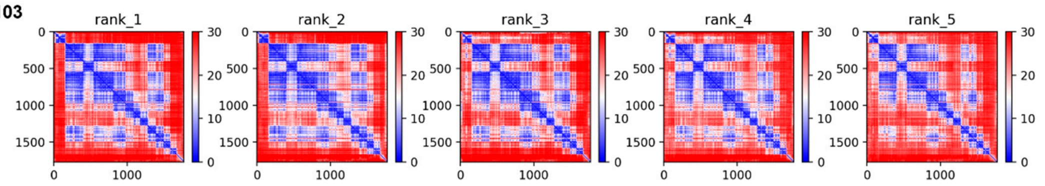

PrtL_{CRL581}

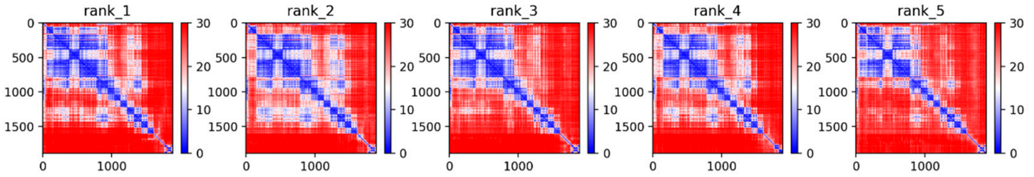

$\text{PrtB}_{NCDO1489}$

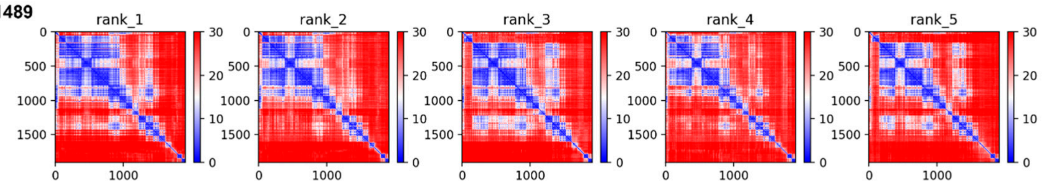

$\text{PrtP}_{NFICC120}$

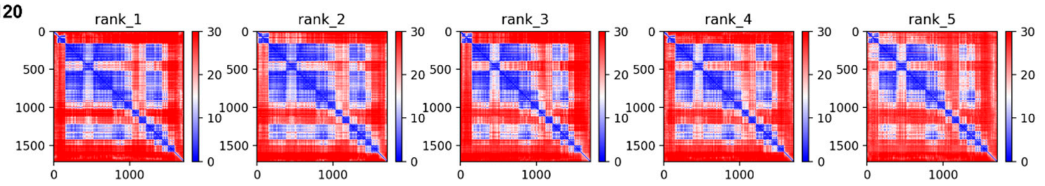

$\text{PrtP}_{NFICC546}$

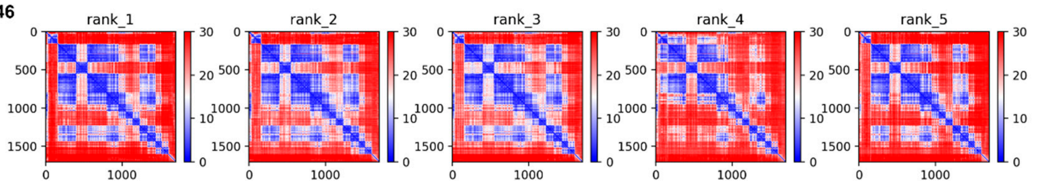

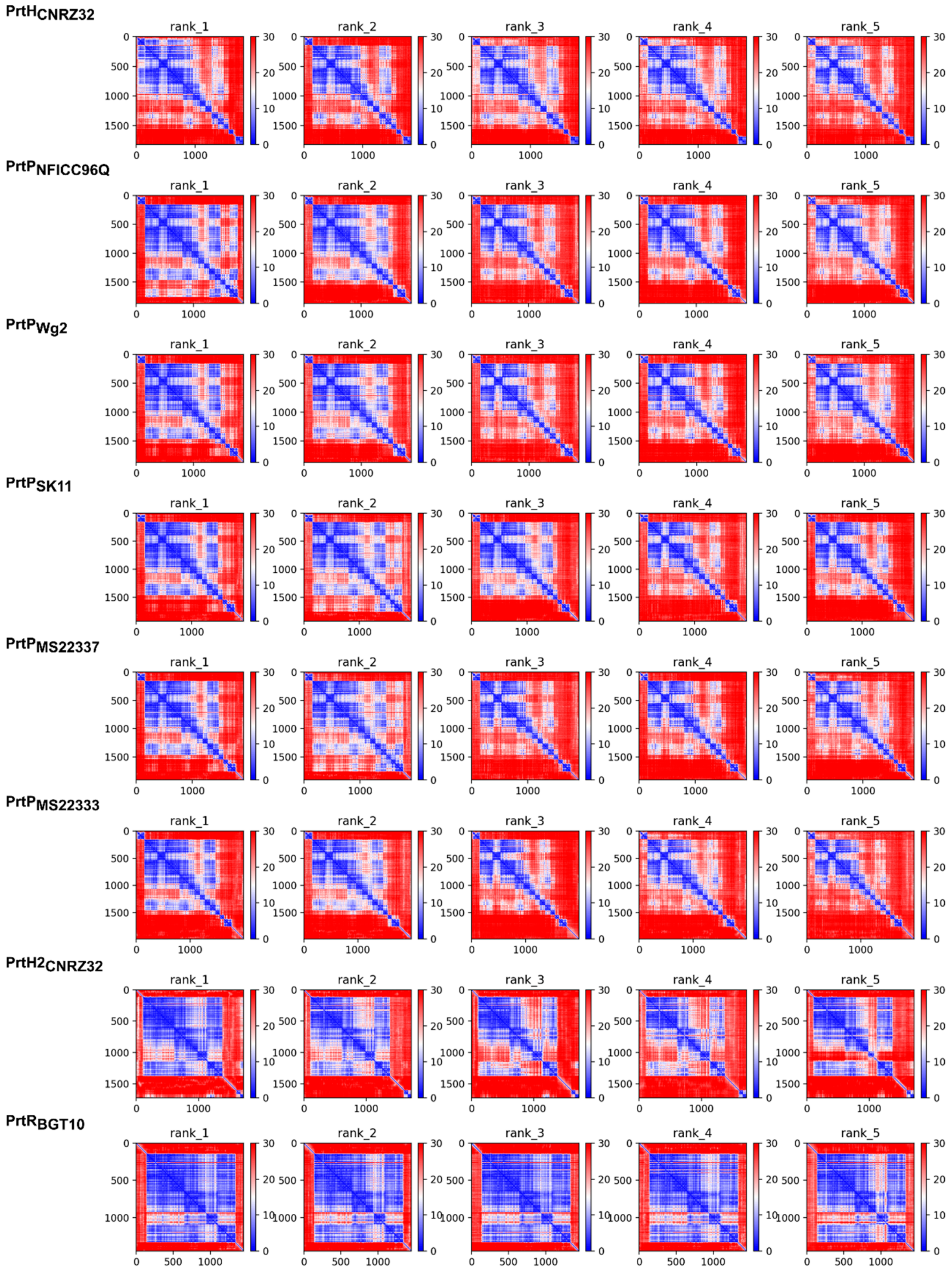
PrtH$_{CNRZ32}$
rank_1 rank_2 rank_3 rank_4 rank_5
PrtP$_{NFICC96Q}$
rank_1 rank_2 rank_3 rank_4 rank_5
PrtP$_{Wg2}$
rank_1 rank_2 rank_3 rank_4 rank_5
PrtP$_{SK11}$
rank_1 rank_2 rank_3 rank_4 rank_5
PrtP$_{MS22337}$
rank_1 rank_2 rank_3 rank_4 rank_5
PrtP$_{MS22333}$
rank_1 rank_2 rank_3 rank_4 rank_5
PrtH2$_{CNRZ32}$
rank_1 rank_2 rank_3 rank_4 rank_5
PrtR$_{BGT10}$
rank_1 rank_2 rank_3 rank_4 rank_5

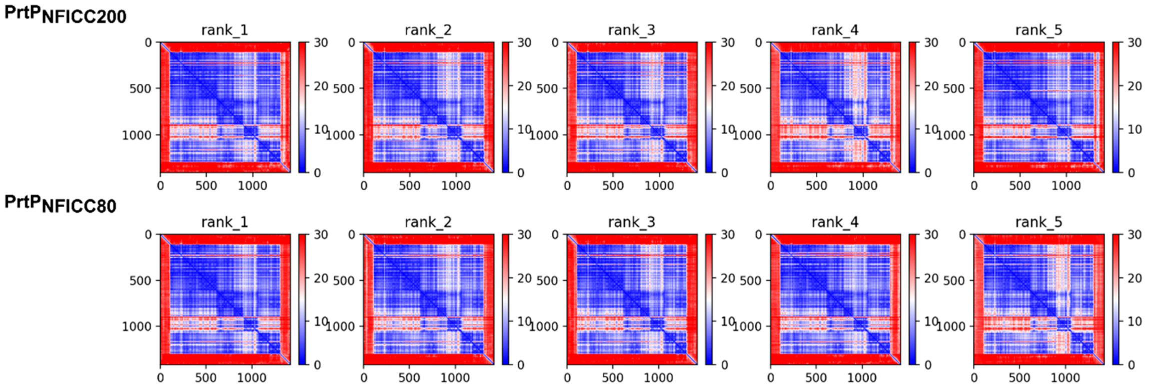

Figure S3. Predicted aligned error (PAE) plots for AlphaFold 2 (AF) models.
PAE provided a distance error for every pair of residues in each AF models of the PrtP homologs. AF had created five structural models for every PrtP homolog that were ranked from 1 to 5. The PAE values were shown as pixels in the heat maps and ranged from 0 to 35 Å. The residue numbers of the proteases were displayed along the horizontal and vertical axes, indicating PAE value for the corresponding residue pair. The prepeptides, with the exception of PrtP$_{NFICC96H}$, were not included in the structure models and thus not in the residue numbers. Low PAE values (< 5 Å) indicated that the relative position of two domains were confidently predicted.

PrtS$_{4F44}$

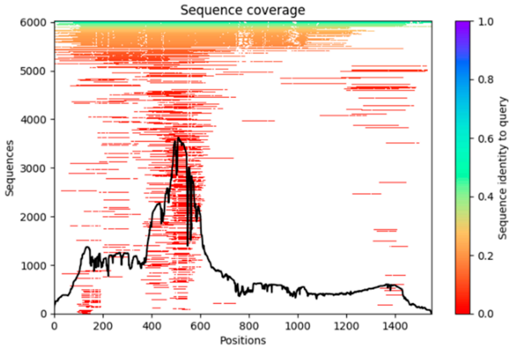

PrtP$_{NFICC96H}$

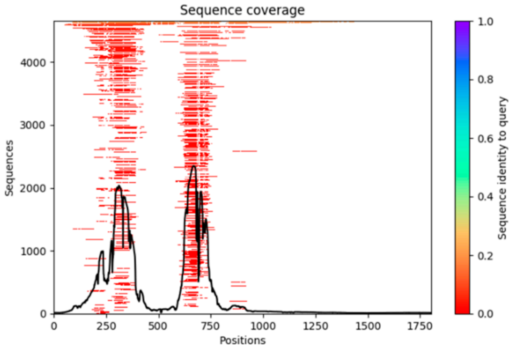

PrtP$_{NFICC96W}$

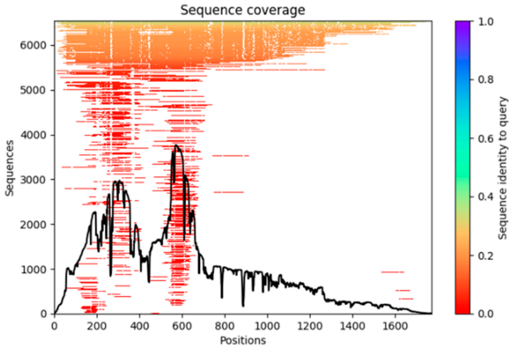

PrtP$_{NFICC103}$

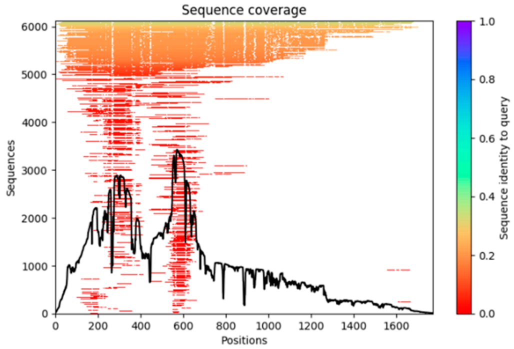

PrtL$_{CRL581}$

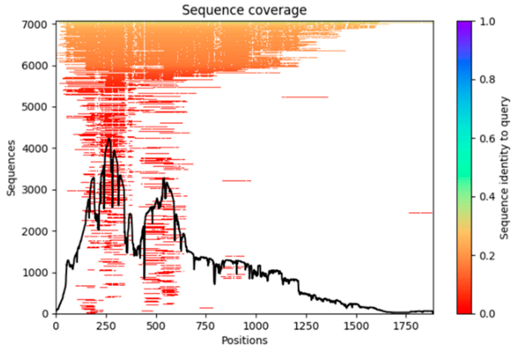

PrtB$_{NCDO1489}$

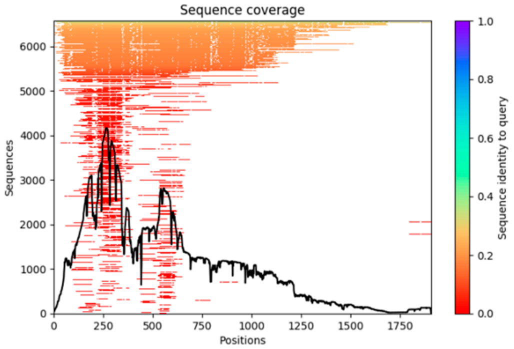

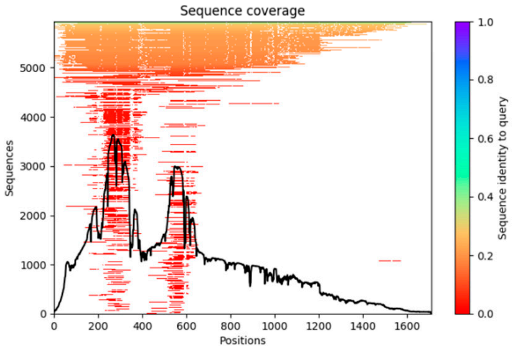

PrtP$_{NFICC546}$

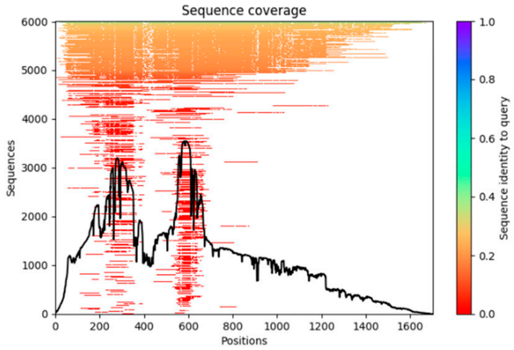

$PrtH_{CNRZ32}$

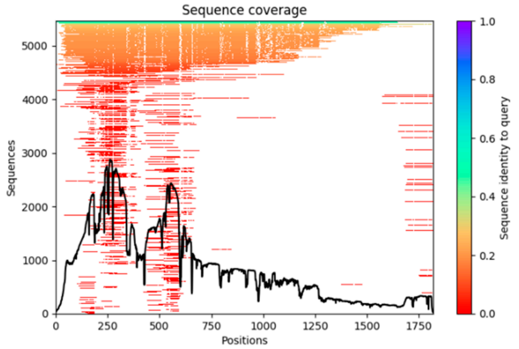

$PrtP_{NFICC96Q}$

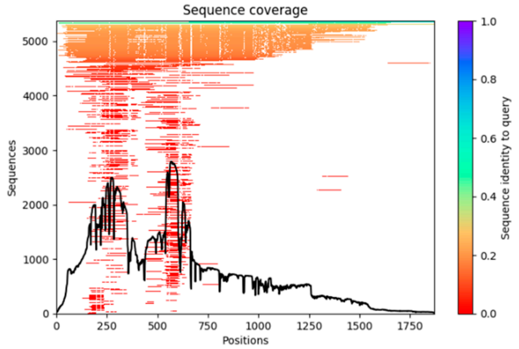

$PrtP_{Wg2}$

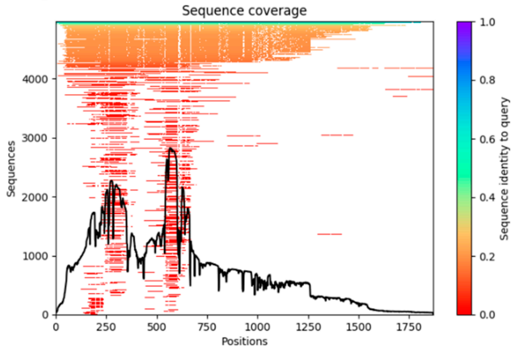

$PrtP_{SK11}$

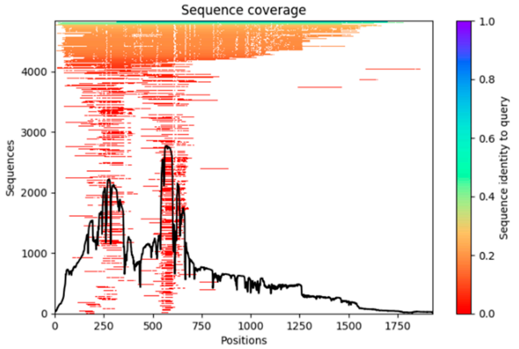

$PrtP_{MS22337}$

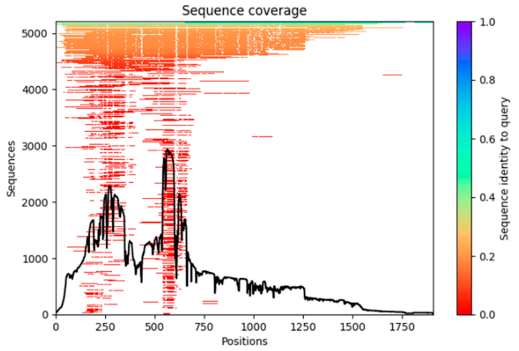

$PrtP_{MS22333}$

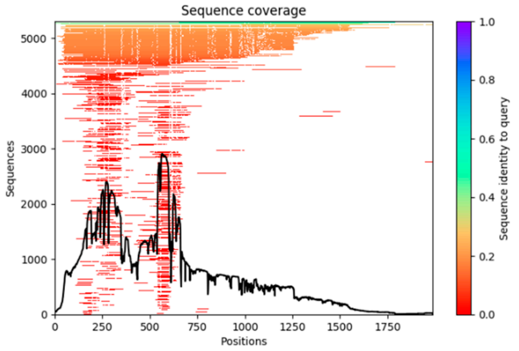

$PrtH2_{CNRZ32}$

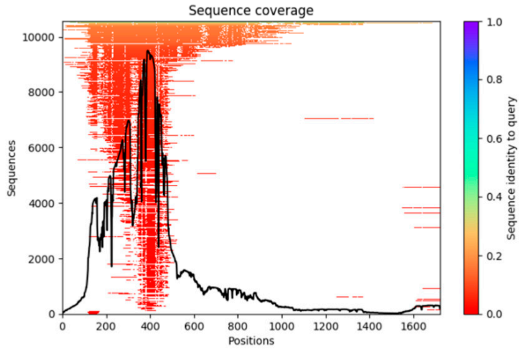

$PrtR_{BGT10}$

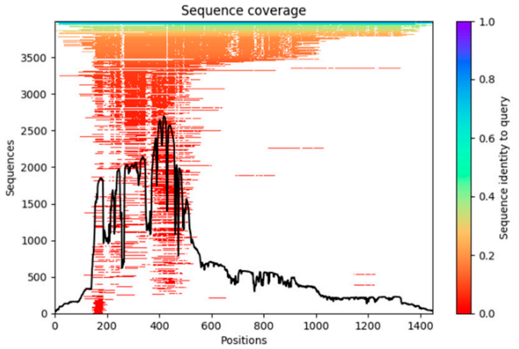

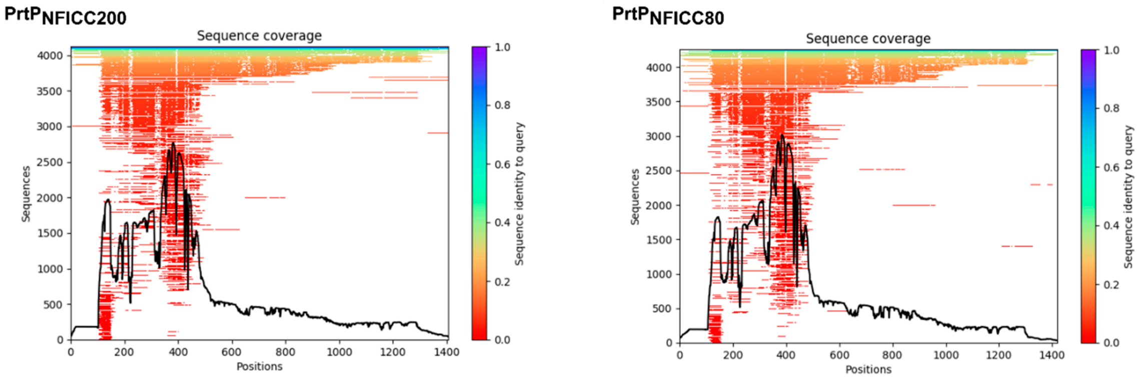

Figure S4. Multiple sequence alignment (MSA) depth for AlphaFold 2 (AF) models.
The plots showed the quality of the MSA used for AF structure modeling. The numbers of protein sequences were shown along the vertical axis, whereas the amino acid position of the query sequence was shown along the horizontal axis. The prepeptides, with the exception of $PrtP_{NFICC96H}$, were not included in the structure models and thus not in the residue numbers. The coverage and identity of the protein sequences to the query sequence was displayed. The plots were output from the ColabFold with the AF implementation, of which the PrtP homologs were used as query sequences.

PrtS$_{4F44}$

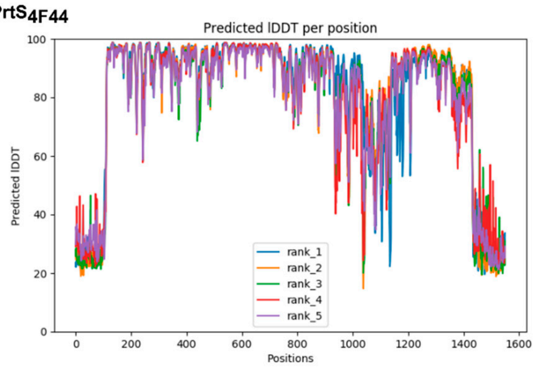

PrtP$_{NFICC96H}$

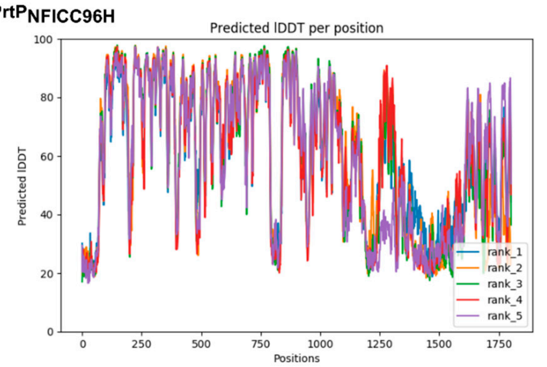

PrtP$_{NFICC96W}$

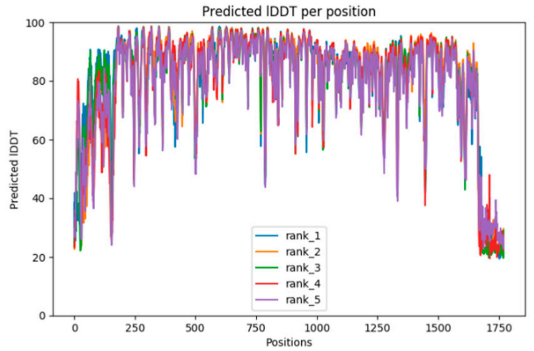

PrtP$_{NFICC103}$

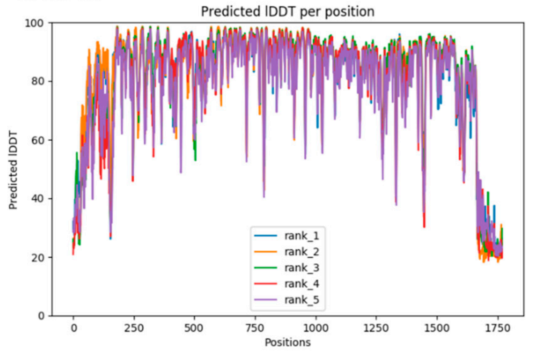

PrtL$_{CRL581}$

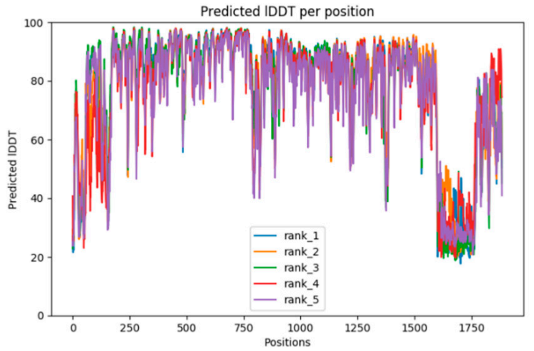

PrtB$_{NCDO1489}$

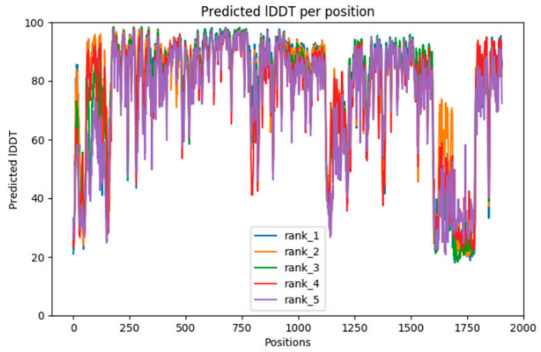

PrtP$_{NFICC120}$

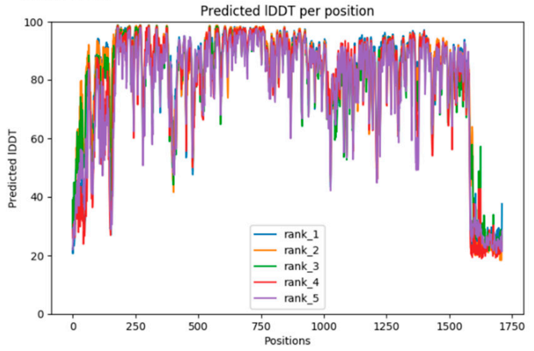

PrtP$_{NFICC546}$

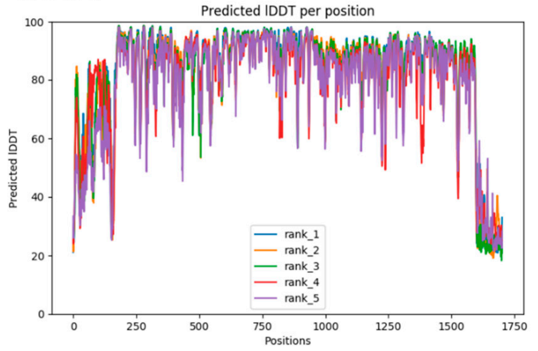

PrtH$_{\text{CNRZ32}}$

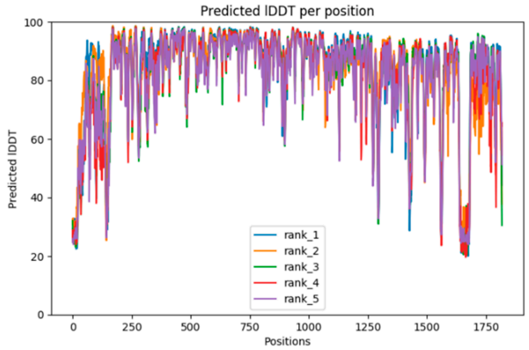

PrtP$_{\text{NFICC96Q}}$

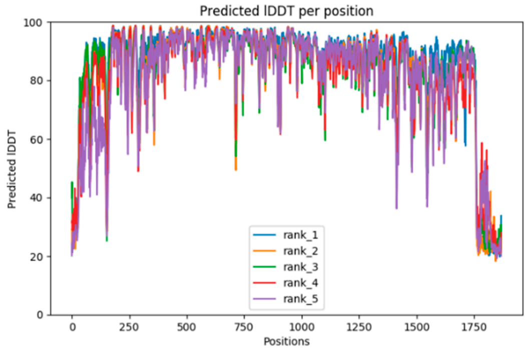

PrtP$_{\text{Wg2}}$

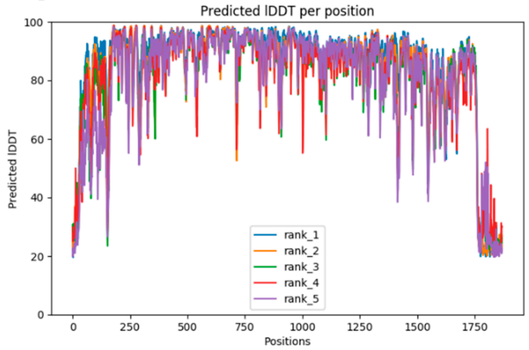

PrtP$_{\text{SK11}}$

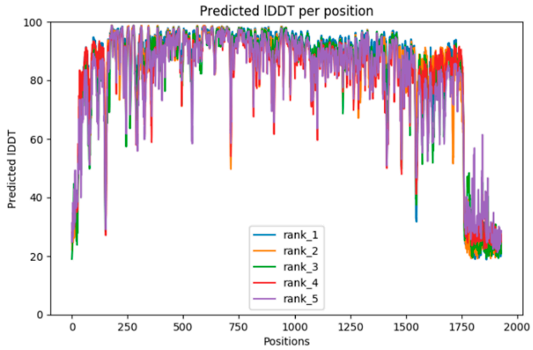

PrtP$_{\text{MS22337}}$

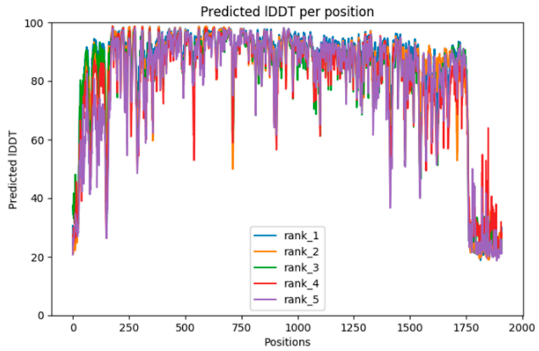

PrtP$_{\text{MS22333}}$

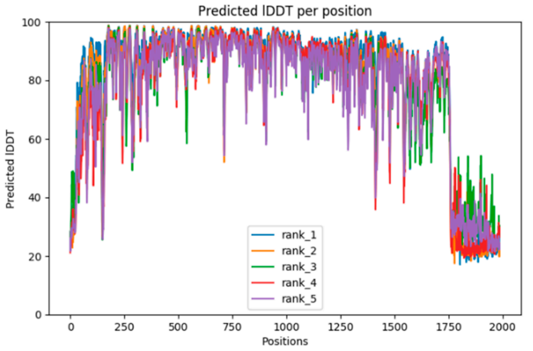

PrtH2$_{\text{CNRZ32}}$

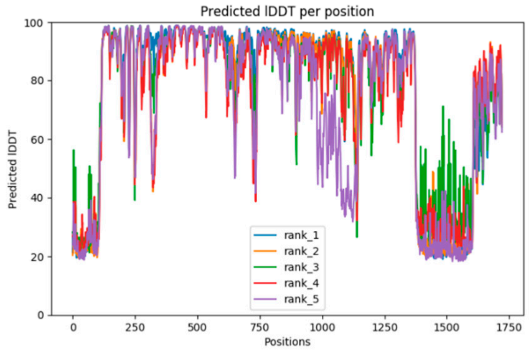

PrtR$_{\text{BGT10}}$

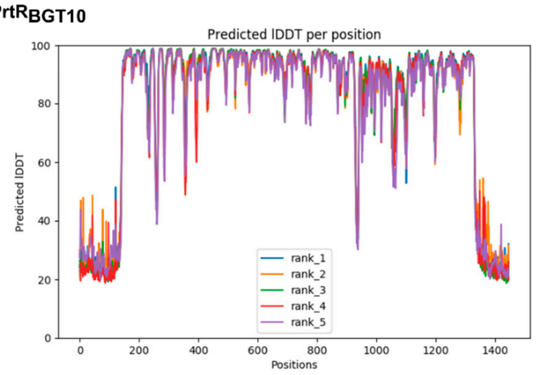

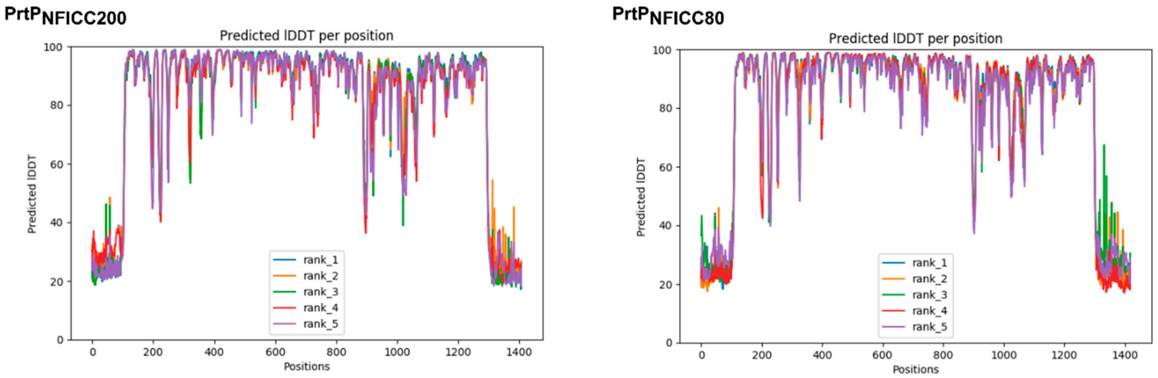

Figure S5. The predicted local distance difference test (pLDDT) for AlphaFold 2 (AF) models.
The plots showed pLDDT (0 – 100) along the vertical axes and the residues numbers of the proteases along the horizontal axes, illustrating the confidence of each amino acid position in each structure model (rank_1 – rank_5). The prepeptides, with the exception of $PrtP_{NFICC96H}$, were not included in the structure models and thus not in the residue numbers. The plots were output from ColabFold with the AF implementation, of which the PrtP homologs were used as query sequences.

A PrtP$_{MS22337}$

Fn	1	2	3	4	5	6	7	8
**1**	-	13	15	13	10	18	16	16
**2**	13	-	7.7	15	12	13	8.4	10
**3**	15	7.7	-	14	4.0	12	9.1	8.0
**4**	13	15	14	-	3.2	8.3	4.4	12
**5**	10	12	4.0	3.2	-	2.0	1.9	3.9
**6**	18	13	12	8.3	2.0	-	3.8	6.9
**7**	16	8.4	9.1	4.4	1.9	3.8	-	8.2
**8**	16	10	8.0	12	3.9	6.9	8.2	-

B ScpC

Fn	1	2	3	4	5	6
**1**	-	11	15	12	15	13
**2**	11	-	16	2.9	13	14
**3**	15	16	-	18	1.6	13
**4**	12	2.9	18	-	15	6.9
**5**	15	13	1.6	15	-	14
**6**	13	14	13	6.9	14	-

Figure S6. Intramolecular comparison of fibronectin type-III-like (Fn) domains.
The structure of the (Fn) domains were superimposed in pairs within PrtP$_{MS22337}$ (A) and ScpC (B). The RMSD values were given for each pair of Fn domains.

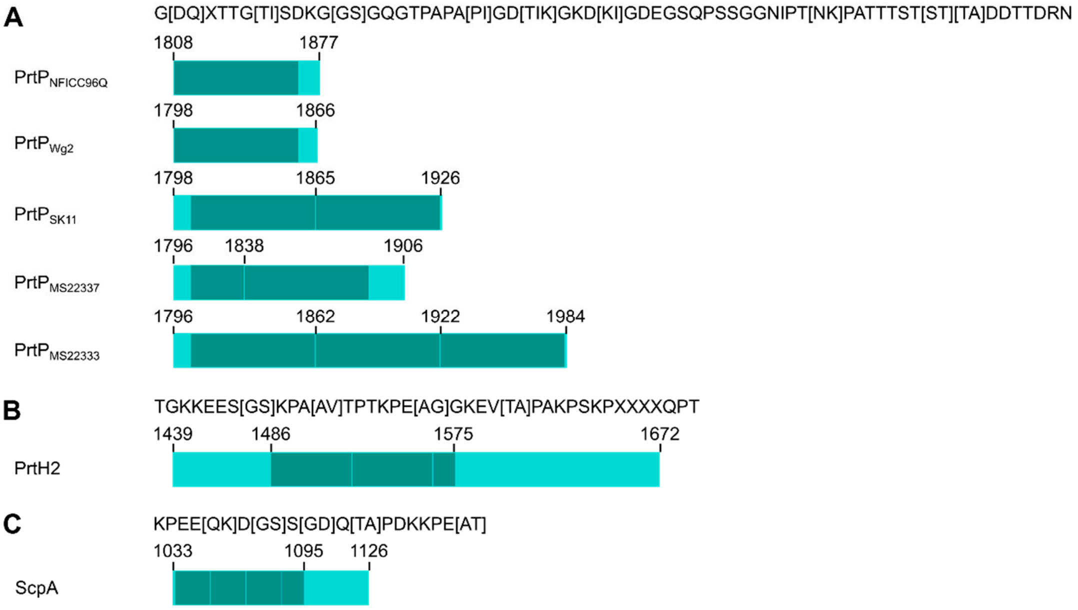

Figure S7. Long tandem repeats in the cell wall spacing (W) domain.
The W-domains of the PrtP homologs were shown as turquoise bar plots, in which darker colored boxes indicated the relative positions of long tandem repeats. The sequence pattern of the tandem repeats were given in top of figure A, B and C.
